# Changes of ambulance departures to assaults during COVID-19 pandemic restrictions

**DOI:** 10.1192/j.eurpsy.2022.1324

**Published:** 2022-09-01

**Authors:** V. Pisl, J. Vevera, J. Volavka

**Affiliations:** 1 Faculty of Medicine and University Hospital in Pilsen, Charles University, Department Of Psychiatry, Plzen, Czech Republic; 2 Faculty of Medicine in Pilsen, Charles University, Department Of Psychiatry, Plzen, Czech Republic

**Keywords:** aggression, assault, lockdown, Covid-19

## Abstract

**Introduction:**

Restrictions related to COVID-19 may affect aggressive behaviour. Increased incidence of gender-based, domestic, and intimate-partner violence was expected during the pandemic, however, retrospective analyses yielded contradicting results.

**Objectives:**

Examine changes in frequency of assaults caused by pandemic restrictions, including separate analysis for male and female assault victims, for residential and non-residential location of assaults and for assaults related to domestic violence.

**Methods:**

Weekly number of ambulance departures to injuries secondary to assaults in the Pilsen region, Czechia, during the COVID lockdown was compared to records from the three previous years using ANOVA and post hoc t-tests. Further, multilinear regression was used to model weekly number of ambulance departures between 1^st^ January 2017 and 30^th^ April 2021 based on presence of pandemic national emergency state, time, and seasonality.

**Results:**

During pandemic lockdown, ambulance departures to assaults dropped by 43% compared to equivalent periods of the three previous years. The decrease was notable specifically among departures to male victims and to assaults in non-residential areas, with only small decrease observed for female victims and assaults related to domestic violence and no change found in frequency of assaults happening at home.

**Conclusions:**

Lockdowns and restrictions of public life were associated with a decreased incidence of violent assaults. While the incidence decreased especially in males and in those assaulted outside of their homes, we found no support for an increase in domestic or gender related violence. Pandemic restrictions may serve as a protective rather than a risk factor for assaults.

**Disclosure:**

No significant relationships.

